# Monitoring and feedback using data in transfusion medicine: A study of practices from the BEST Collaborative

**DOI:** 10.1111/trf.70316

**Published:** 2026-06-22

**Authors:** Ryan A. Metcalf, Allison Mo, Sandra K. White, Gustavo C. Duarte, Nancy M. Dunbar, Katie Hands, Anil K. Panigrahi, Jessica L. Poisson, Nabiha H. Saifee, Hua Shan, Erica M. Wood, Michael F. Murphy, Simon J. Stanworth

**Affiliations:** ^1^ Department of Pathology University of Utah Salt Lake City Utah USA; ^2^ ARUP Laboratories Salt Lake City Utah USA; ^3^ Transfusion Research Unit, School of Public Health and Preventive Medicine Monash University Melbourne Australia; ^4^ Monash Haematology Monash Health Melbourne Australia; ^5^ New Zealand Blood Service Auckland New Zealand; ^6^ Dartmouth Hitchcock Medical Center Lebanon New Hampshire USA; ^7^ Scottish National Blood Transfusion Service, Ninewells Hospital Dundee UK; ^8^ Department of Anesthesiology Stanford University School of Medicine Stanford California USA; ^9^ Department of Pathology Stanford University School of Medicine Stanford California USA; ^10^ Duke University Hospital Durhan North Carolina USA; ^11^ Department of Laboratory Medicine and Pathology Seattle Children's Hospital Seattle Washington USA; ^12^ NHS Blood and Transplant Oxford University Hospitals NHS Trust Oxford UK; ^13^ Blood & Transplant Research Unit (Data‐Enabled), Radcliffe Department of Medicine, University of Oxford Oxford UK

## Abstract

**Background:**

It is imperative that health systems monitor transfusion practice quality and implement improvements when evidence‐based practice gaps are identified. Despite the importance of data‐driven quality activities, little is known about approaches employed by hospitals. This study aimed to describe strategies used by hospitals to acquire, analyze, and apply transfusion‐related quality data.

**Study Design and Methods:**

A pre‐piloted survey was developed by the core team, and Biomedical Excellence for Safer Transfusion (BEST) Collaborative attendees of meetings were invited to participate. Topics spanned five domains: hospital characteristics, data acquisition, quality metric prioritization, data analysis/visualization, and feedback processes. Responses were summarized to identify patterns and variation across institutions.

**Results:**

Nine hospitals of varying size, specialty mix, and transfusion volumes from three continents participated. Most transfusion services acquired transfusion data from both the electronic health record and the laboratory information system (6/9, 67%). Although all sites tracked the number of transfusions, there was wide variation in other metrics prioritized. Transfusion appropriateness was prioritized by 7 of 9 centers, but few reported monitoring antifibrinolytic agent use (2/9, 22%), anemia management (4/9, 44%), and consent (2/9, 22%). All hospitals performed some form of data analysis, but chart/table types and functionalities differed. Feedback was typically provided by transfusion medicine specialists or hematology, but frequency and means differed between sites.

**Discussion:**

This descriptive study highlights significant variation in how selected hospitals use data to monitor transfusion practices and provide feedback. There are many opportunities to enhance optimal uses of data to deliver effective quality improvement.

**Abbreviations**
AABB: Association for the Advancement of Blood and Biotherapies
BEST: Biomedical Excellence for Safer Transfusion
ICTMG: International Collaboration for Transfusion Medicine Guidelines
ISBT: International Society of Blood Transfusion
NICE: National Institute for Health and Care Excellence
SHOT: Serious Hazards of Transfusion
WHO: World Health Organization

## 1 INTRODUCTION

Transfusion teams have a core responsibility to ensure that blood transfusions, which have challenges of supply and risks to recipients, as well as costs, are used appropriately and for evidence‐based clinical indications.^1^, ^2^, ^3^ Monitoring and feedback to prescribers across multiple clinical settings and patient populations is therefore an essential activity of transfusion medicine services and patient blood management programs. However, there is considerable uncertainty in how this is done and delivered in contemporary practice, including how different hospital systems use data to track and ensure the effective implementation of good transfusion practice. Although approaches to data collection and monitoring have traditionally involved manual processes and audits, there is interest in the use of electronic systems with automated capabilities.^4^

Many national and international organizations promote and define the adoption of best practices, such as the Association for the Advancement of Blood and Biotherapies (AABB), the International Collaboration for Transfusion Medicine Guidelines (ICTMG), and the International Society of Blood Transfusion (ISBT). Quality metrics considered pertinent may also be developed from evidence‐based guidelines,^1^, ^2^, ^3^ standards,^5^, ^6^, ^7^ regulations,^8^ as well as national recommendations, such as in England, for which a core list of key national quality standards exist.^9^ Examples of these and additional sources from which quality metrics may be derived are provided in Table 1.^10^, ^11^, ^12^, ^13^, ^14^, ^15^, ^16^, ^17^

**TABLE 1:** Example sources with requirements or standards for metrics.

Source	Specific to country?	Required or voluntary?	Focused summary of metrics/monitoring/standards
US Code of Federal Regulations^8^	US	Required	Review the quality, necessity, and appropriateness of healthcare services provided (e.g., transfusion practices and adverse outcomes).
US CDC National Health Safety Network^10^	US	Voluntary	Hemovigilance module for adverse event reporting
National Institute for Health and Care Excellence (NICE)^17^	England	Required	Statement 1: People with iron‐deficiency anemia who are having surgery receive iron supplementation before surgery. Statement 2: Adults who are having surgery and expected to have moderate blood loss receive tranexamic acid. Statement 3: People are clinically reassessed and have their hemoglobin levels checked after each unit of red blood cells they receive, unless they are bleeding or are on a chronic transfusion program. Statement 4: People who may need or who have had a transfusion are given verbal and written information about blood transfusion.
Serious Hazards of Transfusion (SHOT)^11^	UK	Required	Review appropriate use of blood components, transfusion errors, management of transfusion reactions, and treatments based on reaction type.
Australian Commission on Safety and Quality in Healthcare^12^	Australia	Required	Blood Management Standard Optimizing/conserving patient's own blood Document blood management information Prescribing and administering blood products Reporting adverse blood management events Traceability and availability of blood
New Zealand Transfusion Medicine Handbook^13^	New Zealand	Required	Guide to good clinical practice Examples: prescribing blood components, informed consent, blood stock management, etc.
New Zealand National Haemovigilance Programme^14^	New Zealand	Required	Organized surveillance procedures related to serious or unexpected events or reactions in donors or recipients.
Dutch Hospital Survey^15^	The Netherlands	Required	Ability to generate data for indicators Indication for RBC transfusion Indication and measurement of effect of platelet transfusions Traceability of transfused blood components
International Society for Blood Transfusion (ISBT)	NA	Voluntary	Broad categories: Patient Services (e.g., turnaround time, crossmatch‐to‐transfusion ratio, etc.) Quality management (e.g., serious adverse events)
World Health Organization (WHO) Patient Blood Management^7^	NA	Voluntary	Numerous metrics relevant to PBM. Broad categories include: Presence of guidelines (treatment of iron‐deficiency anemia, minimizing blood loss, evidence‐based transfusions) Outcome metrics (associated with iron deficiency, blood transfusion amounts and appropriateness, iron studies performed, anemia treatment, pregnant women screened, use of antifibrinolytic agents, use of blood recovery systems)
AABB PBM Standards^5^	NA	Voluntary	Anemia program utilization. Iron and micronutrient deficiency identification and management in the outpatient transfusion setting. Bloodless program enrollment/evaluation of effectiveness. Blood use; appropriateness Single‐unit Red Blood Cell transfusion practice performance metric. Blood wastage and discard. Transfusion index and transfusion probability. Crossmatch‐to‐transfusion ratio. Blood administration policy compliance. Deviations. Transfusion reactions. Informed consent for blood transfusion. Massive transfusion/massive hemorrhage protocol use. Use of intraoperative blood recovery equipment and quality control. Blood infusion equipment and warmer(s) maintenance program (annually).
Society for the Advancement of Patient Blood Management^6^	NA	Voluntary	Blood use monitored by provider, clinical service, case type or procedure as well as hospital‐wide. Use of clinical strategies to treat anemia and minimize blood loss is monitored. Data are analyzed to identify potential areas for improvement. Blood and blood component transfusions evaluated using metrics that allow comparison of blood utilization with other institutions. Compliance with patient blood management clinical protocols is monitored. The hospital has a process to assess compliance with blood administration policies and report deviations, adverse events (including avoidable transfusions). The impact of patient blood management and transfusion practice on clinical outcomes.

Relevant data for metrics need to be acquired from reliable sources, and then organized and (ideally) validated prior to local analysis. Following analysis, teams may generate visualizations to provide insights into practice trends and the impact of implemented interventions. Finally, and importantly, changes in transfusion prescribing behavior require feedback of data to relevant clinicians and health care professionals. At present, we have little insight into many elements of these processes, starting with which metrics are prioritized between hospitals and transfusion teams. How transfusion teams perform the critical element of quality improvement feedback remains largely unexplored. The value of interactive quality dashboards to visualize practice performance and provide feedback that have been reported by some hospitals remains unclear.^18^, ^19^, ^20^, ^21^, ^22^

With this background in mind, the aim of this descriptive study was to present more granular information on the approaches for data collection, monitoring, and feedback at selected hospitals. This study would inform further projects in the broad area of quality improvement and feedback to advance our understanding of how data can be more effectively used and to identify opportunities for and to inform future studies for optimizing transfusion practice.

## 2 METHODS

### 2.1 Participants/site selection

A study to describe how selected hospitals acquire, organize, and use data to monitor transfusion practice and drive quality improvements was developed. Institutional review board management was contacted and determined that a formal submission for exemption was not required given that patient‐level data were not collected and the survey captured the programmatic approaches of each site rather than information about individual respondents. Sites were recruited following presentations at a Biomedical Excellence for Safer Transfusion (BEST) Collaborative biannual meeting (conferences 68 and 69). Meeting attendees were asked to contact the lead investigators (RAM, SJS) by email to express interest regarding participation.

### 2.2 Survey development and administration

Members of the working group drafted and piloted an initial survey form containing key domains, each of which included multiple questions. Following iterative pilots, the final survey questionnaire was administered from May through July 2025 to those expressing interest via email as an attached document (Microsoft Word, Redmond, WA). The documents were filled out manually by respondents and returned to the corresponding author.

The five key domains of interest explored in the survey were study site characteristics, data acquisition, clinical transfusion metrics considered important, data analysis and visualization, and use of the synthesized data to provide feedback with the aim of driving improvements in practice. The survey fields of entry included a range of response options, including binary responses (e.g., yes/no), a list of “drop down” options, and free‐text narrative descriptions. A full copy of the survey is provided in Appendix S1.

### 2.3 Survey responses and data

Survey responses were collated, reviewed, and summarized in tables, with each site given a number to maintain anonymity. The response data were summarized by providing qualitative information, with identification of patterns, variability, or perceived gaps.

Information from survey responses was reviewed and interpreted to provide an impression of how participating hospitals from various countries employ data acquisition and analysis to understand and enhance institutional transfusion practice quality.

## 3 RESULTS

### 3.1 Hospital characteristics

Nine hospitals expressed interest and participated in this descriptive study (Table 2). The co‐authors of this manuscript provided responses for their respective sites. Three continents were represented, including Oceania (2 sites), Europe (2 sites), and North America (5 sites). A majority of centers reported treating patients of all ages and providing transfusion services for a variety of complex patient populations. The size of participating institutions, as measured by the number of hospital beds, varied, as did the number of patient discharges and surgeries performed per year. Numbers of transfusions of each blood component also varied by institution.

**TABLE 2:** Hospital characteristics.

Characteristic	Overall	1	2	3	4	5	6	7	8	9
Populations served										
Adult	89% (8/9)	Yes	Yes	Yes	Yes	No	Yes	Yes	Yes	Yes
Pediatric	89% (8/9)	Yes	Yes	Yes	Yes	Yes	Yes	No	Yes	Yes
Neonate	89% (8/9)	Yes	Yes	Yes	No	Yes	Yes	Yes	Yes	Yes
Departments										
Burn surgery	44% (4/9)	No	No	Yes	No	No	No	Yes	Yes	Yes
Cardiovascular surgery	89% (8/9)	Yes	Yes	No	Yes	Yes	Yes	Yes	Yes	Yes
Critical care	100% (9/9)	Yes	Yes	Yes	Yes	Yes	Yes	Yes	Yes	Yes
Gastroenterology	89% (8/9)	Yes	Yes	Yes	No	Yes	Yes	Yes	Yes	Yes
General medicine	100% (9/9)	Yes	Yes	Yes	Yes	Yes	Yes	Yes	Yes	Yes
Hematology/oncology	100% (9/9)	Yes	Yes	Yes	Yes	Yes	Yes	Yes	Yes	Yes
Neurosurgery	100% (9/9)	Yes	Yes	Yes	Yes	Yes	Yes	Yes	Yes	Yes
Obstetrics	89% (8/9)	Yes	Yes	Yes	Yes	No	Yes	Yes	Yes	Yes
Orthopedic surgery	100% (9/9)	Yes	Yes	Yes	Yes	Yes	Yes	Yes	Yes	Yes
Otolaryngology	89% (8/9)	Yes	Yes	Yes	No	Yes	Yes	Yes	Yes	Yes
Solid organ transplant	78% (7/9)	Yes	Yes	No	No	Yes	Yes	Yes	Yes	Yes
Stem cell transplant	100% (9/9)	Yes	Yes	Yes	Yes	Yes	Yes	Yes	Yes	Yes
Trauma	89% (8/9)	Yes	Yes	Yes	Yes	No	Yes	Yes	Yes	Yes
Urology	89% (8/9)	Yes	Yes	Yes	No	Yes	Yes	Yes	Yes	Yes
Number of beds										
1–500	22% (2/9)	1–500	>1000	501–1000	501–1000	<500	>1000	501–1000	>1000	>1000
501–1000	33% (3/9)									
>1000	44% (4/9)									
Annual number of discharges										
<50,000	50% (4/8)	<50,000	<50,000	NA	>125,000	<50,000	50,000‐125,000	<50,000	50,000‐125,000	>125,000
50,000‐125,000	25% (2/8)									
>125,000	25%(2/8)									
Annual number of surgeries										
<30,000	29% (2/7)	<30,000	30,000‐	NA	30,000‐60,000	<30,000	>60,000	NA	>60,000	>60,000
30,000–60,000	29% (2/7)		60,000							
>60,000	43% (3/7)									
Number of RBC transfusions annually										
<10,000	33% (3/9)	<10,000	>20,000	<10,000	10,000‐20,000	<10,000	10,000‐20,000	10,000‐20,000	>20,000	>20,000
10,000‐20,000	33% (3/9)									
>20,000	33% (3/9)									
Number of platelet transfusions annually										
<2500	33% (3/9)	<2500	>5000	<2500	<2500	2500‐5000	2500‐5000	>5000	>5000	2500‐5000
2500‐5000	33% (3/9)									
>5000	33% (3/9)									
Number of plasma transfusions annually										
<2500	44% (4/9)	<2500	>5000	<2500	<2500	<2500	2500‐5000	>5000	>5000	2500–5000
2500‐5000	22% (2/9)									
>5000	33% (3/9)									
Number of cryoprecipitate transfusions annually										
<500	22% (2/9)	500–1000	>1000	<500	500–1000	<500	>1000	>1000	>1000	>1000
500–1000	22% (2/9)									
>1000	56% (5/9)									

### 3.2 Data acquisition and organization

In terms of data sources used to evaluate transfusion practices, most centers extract data from both the electronic health record (6/9, 67%) and the blood bank laboratory information system (8/9, 89%) (Table 3). Data extraction was usually performed using a combination of manual and automated processes (6/9, 67%). For automated data extractions, the frequency of updates on transfusion usage varied from daily to monthly or “as needed”. Four centers reported use of an electronic querying approach, with three of these specifically using Structured Query Language (SQL), a standardized approach to electronically manipulate a relational database.

**TABLE 3:** Data acquisition and organization.

Element	1	2	3	4	5	6	7	8	9
Sources of data:									
EHR	No	Yes	No	NA	Yes	Yes	Yes	Yes	Yes
LIS	Yes	Yes	Yes	NA	Yes	Yes	Yes	Yes	Yes
Other^a^	Yes	No	No	NA	No	No	No	No	No
EHR and LIS integrated or linked	Linked	Linked	N/A^b^	Linked	Linked	Linked	Linked	Linked	Linked
Interface linkage validated	Yes	Yes	N/A^b^	Yes	Yes	Yes	Yes	Yes	Yes
Data acquisition automated	Yes	Yes	Yes	Yes	Yes	Yes	Yes	Yes	Yes
Data acquisition manually	Yes	Yes	No	No	Yes	No	Yes	Yes	Yes
Validated	NA	Yes	Yes	NA	Yes	Yes	Yes	Yes	Yes
Updated automatically	Yes	Yes	Yes	Yes	Yes	Yes	Yes	No	Yes
Updated manually	Yes	Yes	No	No	No	No	Yes	Yes	Yes
Frequency of updates	As needed	Monthly	Monthly	Daily	Daily	Daily	Daily	Weekly	Daily, monthly, yearly, as needed
Query type	Manual	Manual, Tableau	Dashboard	SQL	SQL	Electronic query	SQL	Manual	Automated and manual

Abbreviations: EHR, electronic health record; LIS, laboratory information system; NA, not available; SQL, structured query language.

^a^ Manually populated spreadsheet.

^b^ Data collected for metrics include transfusion data from the LIS and clinical information from coding data that are collected and stored at the national level.

### 3.3 Metrics reported

There was wide variation in the types of metrics prioritized for analysis and reporting (Table 4). As expected, 100% of respondents reported evaluating numbers of transfusions administered. Most centers reported on transfusion appropriateness, such as guideline adherence (7/9, 78%). It was less common for sites to evaluate metrics related to antifibrinolytic use (2/9, 22%), patient consent for blood (2/9, 22%), and the diagnosis and management of anemia (4/9, 44%). There was wide variation in availability or extraction of data linked to other prioritized metrics, apart from blood wastage, which most reported was tracked (7/9, 78%).

**TABLE 4:** Quality metrics prioritized.

Metric	1	2	3	4	5	6	7	8	9
Transfusion amounts	X	X	X	X	X	X	X	X	X
Transfusion appropriateness		X		X	X	X	X	X	X
C:T ratios		X	X		X		X	X	
Anti‐fibrinolytics use						X	X		
Consent						X			X
Anemia management			X			X	X		X
Transfusion reactions	X				X		X		X
MTP/Emergency release	X						X		
Other internal blood bank metrics	X			X			X		X
Inventory	X		X				X		
Traceability						X			X
Wastage	X	X	X	X	X		X		X

Abbreviations: C:T, crossmatch:transfusion; MTP, massive transfusion protocol.

### 3.4 Analysis and visualization

All sites reported that they performed some form of analysis of the data they obtained (Table 5). There was significant variation in descriptions of analyses performed, including the use of tables and visualizations (i.e., chart types). Nearly all sites reported the use of a data dashboard, but again, the levels of complexity and design varied considerably. Most of these were interactive for the user (5/9, 56%) and had some sort of filtering functionality or ability to zoom to view metrics at hierarchical levels within a health system (7/9, 78%). It was common for transfusion medicine specialists and patient blood management professionals to routinely review the data analyses and visualizations.

**TABLE 5:** Analysis and visualization.

Element	1	2	3	4	5	6	7	8	9
Analysis performed	Yes	Yes	Yes	Yes	Yes	Yes	Yes	Yes	Yes
Description	Aggregate totals, highlighted quality metrics	Monthly tracking line charts	Trending of metrics over time	Reports generated to hospital departments using blood	Wastage, C:T ratio, transfusion volume	PBM with highlighted metrics, consent, aggregate totals	Dumbbell charts, scatterplots, heatmaps, line graphs, Pareto charts, stacked bar charts, violin plots, etc.	Data pull using threshold and exclusion criteria followed by clinical case review, bar charts, inappropriate transfusions by floor/unit	Tables, charts, trends over time and comparisons with national benchmarks, manual audits
Analyst	Blood Bank Medical Director	Data engineer, TSO	Hospital transfusion and blood banking teams	PBM team	Lab analysts	Hospital data management group	PBM team	Pathology & Clinical Lab IT team	IT, PBM working group members, hematology or transfusion team
Dashboard	Yes	No	Yes	Yes	Yes	Yes	Yes	No	Yes
Interactive	No	NA	Yes	Yes	Yes	No	Yes	NA	Yes
Automatic	No	NA	Yes	Yes	Yes	Semi	Yes	NA	Yes
Zoom/filter	Some	NA	Yes	Yes	Yes	Yes	Yes	NA	Yes
Charts/tables	Yes	Yes	No	No	No	Yes	Yes	Yes	Yes
Interactive	No	Yes	NA	NA	NA	No	Yes	No	Yes
Automatic	No	Yes	NA	NA	NA	Semi	Yes	No	Yes
Zoom/filter	Some	Yes	NA	NA	NA	Yes	Yes	No	Yes
First reviews data	Blood Bank Medical Director	Data engineer, TSO	Hospital transfusion team	PBM team	Hospital transfusion team	Hematology consultant	Transfusion medicine/PBM	PBM team	Hospital transfusion team, blood management committee

Abbreviations: C:T, crossmatch:transfusion; IT, information technology; PBM, patient blood management; TSO, transfusion safety officer.

### 3.5 Feedback to clinicians

The frequency of feedback to clinicians varied from ad hoc to short intervals (e.g., weekly) to relatively long intervals (e.g., annual cadence) (Table 6). This feedback was typically provided by transfusion medicine and patient blood management team members. Recipients of feedback included physicians in various practice settings, transfusion committee members, leaders of physician groups such as division chiefs, and leaders over the broader health system. Mechanisms of feedback included emails and meeting environments ranging from small numbers of people to grand rounds presentation settings. The perception of the impact of feedback to improve practice varied substantially, from no clear change to evidence of improved guideline adherence, cost savings, reduced adverse events, and increased rates of anemia screening.

**TABLE 6:** Feedback to clinicians.

Question	1	2	3	4	5	6	7	8	9
Frequency of feedback	As needed for MTP utilization	As needed for wastage, monthly for visualizations	Quarterly to transfusion committee on inventory, specific feedback on IDA	Usually monthly, quarterly for cell saver	Blood usage committee—2×/year, transfusion administration metrics for nurse leaders—annually, platelet utilization project	Provided quarterly	Provided quarterly and ad hoc	Weekly	Varies, monthly
Provides feedback	Blood Bank Medical Director	TSO, Medical director of transfusion lab	Consultant hematologist, laboratory manager	PBM team leader	Transfusion safety consultant	Consultant hematologist	PBM leaders, transfusion medicine specialist	TM physician or anesthesiologist	Transfusion nurses, blood bank scientists, hematologists
Feedback provided to	Section leaders	Individual clinicians, transfusion committee	Grand rounds to hospital, department level	Clinical nurse manager, Clinical directors	Attending and nurse leaders for departments and divisions	Hospital division chairs	Physicians, division chiefs, hospital leaders	Unit/floor physician quality leads	Transfusion committee, individual clinical units, blood bank scientist
Feedback description	Email with specific event data	Email to individual clinicians regarding specific over‐ordering	High plasma wastage	–	Broad summary of data and dashboards	Compliance with RBC and platelet thresholds	Quarterly emails, ad hoc data visualizations of performance	Summary bar graphs with clinical context summary and recommendation	Dashboard visualization, reports, education sessions
Feedback impact	Unknown	Targeted education	Wastage of plasma fell below KPI	–	Improvement of transfusion administration and specimen rejection	Improving adherence to Hgb thresholds and single‐unit transfusions	Reduction in transfusions per case mix weighted discharge, cost savings, projected reduction in adverse events	No significant discussion or change so far	Pre‐op anemia‐‐increased uptake of pre‐op screening has further enhanced collaborations with the surgical units

Abbreviations: Hgb, hemoglobin; IDA, iron deficiency anemia; MTP, massive transfusion protocol; PBM, patient blood management; TSO, transfusion safety officer.

## 4 DISCUSSION

The acquisition and use of data is essential to monitor and drive improvements in transfusion practices, but little is known about what hospitals are actually doing in this regard. In our survey, we identified significant variation by site in areas of data acquisition, metric prioritization, analysis, and feedback to health care professionals. It should be noted that the participating sites in this study are likely to be supported by motivated transfusion specialists because of the method used to offer participation in this study through BEST, and hence demonstrate more willingness to engage with quality improvement initiatives. However, we imagine that wider variation in data practices is more likely to be found if the survey was circulated to a wider array of hospitals. The findings underscore many opportunities for improvement and standardization in monitoring and feedback but represent an important starting position to further study ways to identify optimal processes for data collection and analysis that could bridge gaps in effective monitoring, feedback, and benchmarking of transfusion practices.

All sites performed most clinical services and there did not appear to be clear patterns in variation based on site characteristics. Most or all sites prioritized metrics related to blood utilization and the appropriateness of blood use. The former tends to be required by regulatory bodies, while the latter is generally considered important because blood transfusion is a very common intervention that comes with important risks and where it is known that inappropriate use is substantial. Exactly how hospitals evaluate transfusion appropriateness (e.g., guideline adherence) is an important research question which we believe will be best addressed by a dedicated follow up study.

The selection of metrics and their definitions was variable between sites. Several additional metrics were reported as prioritized by at least one site, and many sites did not report prioritizing metrics that other sites considered important. Data sources in healthcare are complex, and the acquisition and analysis of pertinent data for key metrics can present technical and resource challenges. Again, there was variation in how relevant data are acquired and organized by site. Overall, although standards for metrics of the quality of transfusion practice exist, it does not appear common for hospitals to monitor all of them.

Furthermore, dashboards to display information for review by clinicians varied between sites. There are key principles groups may consider when designing a quality dashboard in healthcare,^23^ but little information about how these relate to transfusion practices. When first viewing a dashboard, it may be helpful to have quick views of clearly labeled important summary metrics automatically displayed for users. Interactive functionality to explore high‐priority questions and easily create reports, as well as the ability to jump to different hierarchical levels of a health system to visualize performance, may also be beneficial. We also have a limited understanding of how hospital clinicians perceive and whether they apply data‐driven feedback into their daily practices. It remains unclear if feedback actually improves transfusion practices across a hospital.^4^, ^18^

This study has limitations. As mentioned, one clear limitation is the selection of sites that were motivated to participate in this project through BEST, such that there may be selection bias and the responses may not be representative of the range of sites and practices that exist worldwide. Nevertheless, we might expect that, if anything, there could be greater practice gaps or variation in a more broadly representative sample. The sample size was small, but as a descriptive study, this allowed more detailed reporting for each individual site. Nevertheless, reliance on free‐text survey responses may still limit the capture of certain nuances of institutional practices and the consistency of the data collected.

In conclusion, this descriptive study is an initial step that highlights significant gaps and opportunities in how hospitals use data to monitor and improve practice. We found wide variation among the reporting sites with respect to how relevant data are acquired, analyzed, and applied. There are many avenues for future research, including steps to optimize chart types, the science of feedback and benchmarking, use of data for economic evaluations, and need for qualitative research, including a better understanding of technical and resource barriers (Table 7). There is a need to address concerns if hospitals assume that, although practice could improve, it may be difficult to change, or perceptions that practice may already be optimized in a given area such that it may not be worthwhile to expend energy and resources to validate and analyze the relevant data. This area of practice change is specifically challenging since transfusion is the most performed procedure in hospital systems and many diverse populations of patients receive blood, which means that feedback needs to consider multiple clinical specialties and practitioners. The benefits of moving toward optimal transfusion practices include improved patient outcomes, minimizing many different clinical populations at risk of the impact of blood supply shortages, and a reduction in healthcare‐related costs.

**TABLE 7:** Research needs.

Priorities for future research	Uncertainties to address
Optimal approaches to monitor transfusion appropriateness	The challenges of standardizing approaches and how to accurately measure appropriateness, which will require context. E.g., ability to ascertain clinical patient population (including active hemorrhage) and all pertinent laboratory values.
Optimal benchmarking blood use amounts	What is the validity of tracking transfusion amounts over time using adjustment factors for patient population risk (e.g., case mix index) and number of patients seen in the hospital system?
Clinician perception of feedback	The uncertain engagement by clinicians, with needs for qualitative studies of clinician perception of feedback using structured interviews.
Optimal approaches to visualize metrics	Although it is common for programs to present data in the form of charts, such as bar charts, line charts, 80:20 charts, and more, it is unclear how often charts chosen are the most appropriate for a given circumstance or use case.
Use of data for economic evaluations	What are the optimal approaches to evaluate the costs of implementing changes, and the economic impact of making changes or improvements in clinical practice?
Barriers	What are the technical and resource barriers limiting a hospital program's ability to obtain, visualize, and use data metrics to drive practice improvements?

## CONFLICT OF INTEREST STATEMENT

R.M. reports being a co‐inventor of a data visualization tool for patient blood management, which is not currently commercialized.

## Supporting information


**Data S1.** Appendix survey.

**Data S2.** CROSS_checklist.

**Data S3.** Supplement_Descriptive study.

## Data Availability

The data that support the findings of this study are available from the corresponding author upon reasonable request.
