# Supplementary material for: Monitoring and feedback using data in transfusion medicine: A study of practices from the BEST Collaborative
Source: Transfusion. 2026 Jun 22;66(10):1939–51. doi: 10.1111/trf.70316 (PMC13674114; doi:10.1111/trf.70316)
Supplement: Supplementary file 2 [file TRF-66-1939-s002.docx]

**Supplement**

Below are examples of data visualizations employed by various sites included in the study. These are ordered randomly and do not correspond to the site numbering in the manuscript, because many of the visualizations below are labeled with site information.

Example 1: Advanced data visualization.

Three simultaneous visualizations are filtered to focus on coronary artery bypass graft procedures. The left panel shows elective, urgent, and emergent case types are included and a filter was used to exclude cases with zero intraoperative red blood cell (RBC) transfusions. The top shows a dumbbell chart with postoperative hemoglobin levels (y-axis, blue dots) by surgeon (x-axis) that can be used to identify over-transfusion when hemoglobin values are well above the post-transfusion target of ~7.5-9 g/dL. The bottom left shows a heatmap of cell salvage volume administered in ml (x-axis) by anesthesiologist (y-axis). Because only cases with at least one allogeneic RBC transfusion were included, 0ml of cell salvage could indicate under-use. The bottom right demonstrates variation in cost of transfusions by blood component using this stacked bar chart. Potential cost savings using cell salvage is shown and all cost settings are customizable.

Example 2: Transfusion amounts adjusted for risk and number of patients over time

This bar chart shows number of RBCs transfused to inpatients adjusted for patient complexity (using case mix index, CMI) and the number of inpatients coming through the system (discharges).

Example 3: Distribution of pre-transfusion hemoglobin ranges by specialty

Example 4: Blood Transfusion Dashboard

Example 5: Wastage

Example 6: Transfusion amounts and adverse events

Example 7: Pre-transfusion platelet count

Example 8: Transfusions in hip surgery population

Example 9: Iron deficiency anemia

Example 10: Transfusion amounts and hemoglobin values

Example 11: Transfusion amounts
