# Supplementary material for: Monitoring and feedback using data in transfusion medicine: A study of practices from the BEST Collaborative
Source: Transfusion. 2026 Jun 22;66(10):1939–51. doi: 10.1111/trf.70316 (PMC13674114; doi:10.1111/trf.70316)
Supplement: Supplementary file 3 [file TRF-66-1939-s003.docx]

**BEST study on the use of data to improve practice**

**Aim: to scope the pathways and approaches by which hospital transfusion departments collect data and provide feedback on specific categories of indicators for transfusion practice.**

We all do or should collect and use data:

Transfusion usage

Number of transfusions (perhaps adjusted for complexity and number of patients or patient days)

Adherence to recommendations in local/national/international transfusion guidelines

Analysis and methods of collecting and presenting these data

Share information with clinicians with a premise to improve practices (assuming discrepancy between performance and standard)

But how do we do this ‘on the ground’?

Methods – Survey small group to describe in some detail how data are collected/organized, analyzed, and used for feedback

**Hospital/Health System Characteristics**

| **Hospital/System** | **Response** |
| --- | --- |
| Country |  |
| Patient populations served:   - Adult, pediatric, neonatal - Hematology/oncology, stem cell transplant, trauma, cardiovascular surgery, solid organ transplant, obstetrics, critical care, neurosurgery, urology, orthopedic surgery, burn surgery, otolaryngology, gastroenterology, general medicine - Inpatient, outpatient, home transfusion |  |
| Number of beds |  |
| Number of patient discharges (or admissions) per year |  |
| Number of operating room surgeries per year |  |
| Reporting for multiple hospitals? |  |
| Number of transfusions per year: | |
| RBCs |  |
| Platelets |  |
| Plasma |  |
| Cryoprecipitate |  |
| Whole blood |  |

**Data Acquisition and Organization?**

| **Element** | **Question** | **Response** |
| --- | --- | --- |
| Data acquisition | Description of current process: sources of data, whether/how these flow into a database or are used to create charts manually, who performs?  (See two examples provided below for reference) |  |
|  | Is data acquisition manual, automated/electronic, or a combination? |  |
|  | Is database validated such that it is confirmed to match the source data as intended? (Y/N) |  |
|  | What validation occurs? Please describe.  Example: manual crosschecking a patient subset in your database with corresponding individual patient charts to identify any discrepancies. |  |
|  | Is it updated manually or automatically?  How often is it updated? |  |
|  | How is it queried?  Example: manually, SQL, etc. |  |

**Blood Utilization Metrics**

| **Element** | **Question** | **Response** | **Justification (e.g. guidelines, standards; gaps/opportunities)** | **Data source** |
| --- | --- | --- | --- | --- |
| Describe metrics reported for:   1. Blood component use 2. Blood component use appropriateness | What are the exact metrics you use to track these? Please provide as much detail as possible. |  |  |  |
|  |  |  |  |  |
|  |  |  |  |  |
|  |  |  |  |  |
|  |  |  |  |  |
|  |  |  |  |  |
|  |  |  |  |  |
|  |  |  |  |  |
|  |  |  |  |  |

**Analysis & Visualization**

| **Element** | **Question** | **Response** |
| --- | --- | --- |
| Analysis | What analysis is done?   - For example, analysis may be required to obtain one or more of the metrics you listed above. - Or to create charts/figures. Describe charts/figures you use (e.g. bar charts, run charts, Pareto/80:20 charts, scatterplots, etc). - Or to create tables. - How do you evaluate whether important changes in your metrics occur over time (or not)? | *Provide description here* |
|  | Who performs/provides the analysis? (e.g., internal to transfusion team, IT team, other) |  |
| Visualization (provide examples) | Is it a dashboard or separate bespoke charts/tables? |  |
|  | Does it populate automatically? (Y/N) |  |
|  | Is it interactive, as opposed to static? (Y/N) |  |
|  | If yes, can it be zoomed/filtered? (Y/N) |  |
|  | Who first reviews data (hospital transfusion team or other)? |  |
|  |  |  |

**Feedback to Clinicians** PROVIDE ACTUAL EXAMPLES

| **Question** | **Response** | **Justification/Reasoning** |
| --- | --- | --- |
| How often is feedback provided to clinicians? (Monthly, quarterly, no specified interval, etc.) |  |  |
| Who provides the feedback (job title)? |  |  |
| To whom is feedback provided (e.g. individual clinicians, department/division/section leaders, overall health system leaders, etc.). |  |  |
| Description of feedback provided | *Provide example or description* | *Provide example or description* |
| Briefly describe the impact of feedback |  |  |

**Effectiveness and Future Plans**

| **Question** | **Response** | **Justification/Reasoning** |
| --- | --- | --- |
| Do you have insights/findings on effectiveness? Or how clinicians and end-users like/dislike? |  |  |
| What are your plans for the next 3 years? |  |  |
